# Supplementary material for: Factors Influencing Healthcare Experience of Patients with Self-Declared Diabetes: A Cross-Sectional Population-Based Study in the Basque Country
Source: Healthcare (Basel). 2021 Apr 28;9(5):509. doi: 10.3390/healthcare9050509 (PMC8145886; doi:10.3390/healthcare9050509)
Supplement: Supplementary file 1 [file healthcare-09-00509-s001.zip › healthcare-1186297-supplementary.pdf]

## Supplementary Material

**Table S1:** Model 1–WLS results (detailed). Differences in healthcare experience among patients with self-declared diabetes. The effect of sociodemographic and economic characteristics.

| Variable | Category | Factor 1: INTER Coef. (CI 95%) | Factor 2: NEW Coef. (CI 95%) | Factor 3: SELF Coef. (CI 95%) | OVERALL IEXPAC Coef. (CI 95%) |
|---|---|---|---|---|---|
| Gender Baseline: Women | Men | -0.078 (-0.445,0.290) | 0.231 (-0.115,0.576) | -0.151 (-0.517,0.215) | -0.020 (-0.315,0.274) |
| Age ranges Baseline: 25-44 | 45 to 64 | 0.687 (-2.374,3.749) | 1.714 (-0.367,3.796) | 3.174** (1.019,5.330) | 1.872* (-0.214,3.958) |
| | 64 to 75 | 0.791 (-2.100,3.682) | 1.114 (-0.730,2.959) | 3.826*** (1.880,5.772) | 1.983** (0.067,3.899) |
| | 75 to 89 | 0.424 (-2.467,3.315) | 0.805 (-1.016,2.626) | 3.305** (1.221,5.389) | 1.576 (-0.373,3.524) |
| | 90 or over | -0.410 (-3.182,2.363) | -0.131 (-1.774,1.513) | 1.100 (-0.695,2.894) | 0.215 (-1.599,2.029) |
| Occupation Baseline: Managers I | Managers II | 2.141** (0.493,3.789) | 5.555*** (2.430,8.680) | 5.597** (1.436,9.758) | 4.329** (1.505,7.153) |
| | Intermediate | 0.200 (-1.038,1.438) | -0.599 (-1.433,0.236) | 0.103 (-1.396,1.603) | -0.053 (-1.059,0.953) |
| | Semi-qualified | -1.655 (-7.445,4.134) | 5.356** (1.012,9.701) | -1.132 (-4.669,2.405) | 0.447 (-4.029,4.924) |
| | Non-qualified | 1.203** (0.256,2.151) | 0.972** (0.082,1.861) | 2.784*** (1.807,3.762) | 1.715*** (1.208,2.222) |
| Education Baseline: Primary | Secondary-lower | -3.149 (-8.881,2.582) | 2.461 (-3.248,8.171) | -2.252 (-6.394,1.889) | -1.293 (-6.004,3.418) |
| | Secondary-upper | -1.962 (-6.092,2.167) | -2.410 (-5.327,0.506) | -0.688 (-3.154,1.777) | -1.621 (-4.751,1.508) |
| | Tertiary | -0.051 (-2.999,2.897) | 0.981 (-2.336,4.298) | 0.503 (-3.825,4.831) | 0.432 (-2.859,3.723) |
| Occupation #Age Baseline: Managers I # 25 to 44 | Managers II # 45 to 64 | -2.080** (-4.096, -0.064) | -5.068** (-8.623, -1.512) | -4.671** (-8.972, -0.370) | -3.837** (-6.828, -0.847) |
| | Managers II # 64 to 75 | -2.664** (-4.915, -0.413) | -5.119** (-8.744, -1.493) | -5.754** (-10.170, -1.338) | -4.457** (-7.580, -1.334) |
| | Managers II # 75-89 | -0.372 (-2.426,1.681) | -5.358** (-9.341, -1.375) | -3.119 (-7.435,1.198) | -2.731* (-5.739,0.278) |
| | Managers II # >=90 | 0.359 (-1.289,2.007) | -5.555*** (-8.680, -2.430) | -0.597 (-4.758,3.564) | -1.601 (-4.425,1.222) |
| | Intermediate # 45 to 64 | -1.755 (-4.749,1.239) | 0.889 (-1.603,3.381) | -0.704 (-2.797,1.389) | -0.651 (-2.855,1.552) |
| | Intermediate # 64 to 75 | -1.324 (-3.220,0.572) | 0.528 (-0.881,1.937) | -0.857 (-2.829,1.115) | -0.649 (-2.123,0.825) |
| | Semi-qualified # 45 to 64 | 1.631 (-4.247,7.510) | -5.307** (-9.851, -0.764) | 1.813 (-1.891,5.517) | -0.195 (-4.757,4.367) |
| | Semi-qualified # 64 to 75 | 1.266 (-4.590,7.122) | -4.981** (-9.436, -0.525) | 1.162 (-2.491,4.815) | -0.475 (-5.014,4.063) |
| | Semi-qualified # 75 to 89 | 2.049 (-3.804,7.902) | -5.151** (-9.589, -0.713) | 1.382 (-2.354,5.118) | -0.157 (-4.705,4.390) |
| | Semi-qualified # >=90 | 1.577 (-5.692,8.847) | -4.501** (-8.980, -0.021) | 3.012 (-2.447,8.471) | 0.441 (-5.080,5.963) |

| | | | | | |
|---|---|---|---|---|---|
| | Non-qualified # 45 to 64 | -2.000** | -1.179* | -3.021*** | -2.147*** |
| | | (-3.417, -0.583) | (-2.555,0.196) | (-4.446, -1.596) | (-3.139, -1.156) |
| | Non-qualified # 64 to 75 | -1.705** | -0.920 | -3.305*** | -2.073*** |
| | | (-2.914, -0.496) | (-2.152,0.312) | (-4.539, -2.072) | (-2.869, -1.277) |
| | Non-qualified # 75 to 89 | -1.295** | -0.772 | -2.893*** | -1.733*** |
| | | (-2.566, -0.023) | (-1.983,0.439) | (-4.372, -1.413) | (-2.644, -0.823) |
| Education#Age | Secondary lower # 45 to 64 | 2.431 | -3.087 | 1.741 | 0.675 |
| Baseline: | | (-3.415,8.278) | (-8.894,2.719) | (-2.536,6.019) | (-4.119,5.470) |
| Primary # 25 to 44 | Secondary lower # 64 to 75 | 2.935 | -2.913 | 1.913 | 0.969 |
| | | (-2.832,8.703) | (-8.651,2.825) | (-2.278,6.105) | (-3.772,5.709) |
| | Secondary lower # 75 to 89 | 3.093 | -2.716 | 2.130 | 1.159 |
| | | (-2.668,8.854) | (-8.450,3.018) | (-2.058,6.318) | (-3.576,5.893) |
| | Secondary lower # >=90 | 3.118 | -3.203 | 1.192 | 0.694 |
| | | (-2.752,8.989) | (-8.976,2.571) | (-3.422,5.806) | (-4.142,5.530) |
| | Secondary upper # 45 to 64 | 1.536 | 2.071 | 0.509 | 1.308 |
| | | (-2.758,5.830) | (-1.002,5.143) | (-2.174,3.193) | (-1.942,4.558) |
| | Secondary upper # 64 to 75 | 1.973 | 2.641* | 0.435 | 1.596 |
| | | (-2.213,6.160) | (-0.372,5.654) | (-2.137,3.007) | (-1.588,4.780) |
| | Secondary upper # 75 to 89 | 2.101 | 3.248** | 1.038 | 2.027 |
| | | (-2.089,6.290) | (0.122,6.373) | (-1.638,3.715) | (-1.197,5.252) |
| | Secondary upper # >=90 | 0.010 | 3.474** | -1.963 | 0.237 |
| | | (-4.140,4.159) | (0.523,6.425) | (-4.458,0.533) | (-2.916,3.391) |
| | Tertiary # 45 to 64 | 0.178 | -0.712 | -0.660 | -0.370 |
| | | (-3.028,3.384) | (-4.356,2.931) | (-5.110,3.790) | (-3.800,3.060) |
| | Tertiary # 64 to 75 | -0.744 | -0.136 | -1.656 | -0.909 |
| | | (-4.219,2.732) | (-3.860,3.588) | (-6.348,3.037) | (-4.506,2.687) |
| | Tertiary # 75 to 89 | -1.058 | -0.099 | -1.134 | -0.824 |
| | | (-4.514,2.398) | (-4.055,3.856) | (-5.910,3.642) | (-4.477,2.829) |
| | Constant | 7.988*** | -0.100 | 4.051*** | 4.351*** |
| | | (5.183,10.792) | (-1.772,1.572) | (2.234,5.869) | (2.523,6.178) |
| Goodness-of-fit | R-squared | 0.067 | 0.092 | 0.074 | 0.058 |
| | BIC | 2536.062 | 2453.380 | 2550.006 | 2307.144 |
| Sample size (¥) | | 554.000 | 554.000 | 554.000 | 554.000 |

* p < 0.1, ** p < 0.05, *** p < 0.001; BIC: Bayesian Information Criterion; The presented model is corrected from heteroscedasticity using Eicker–Huber–White standard errors. ¥: There is one missing response for occupation, and has been excluded for the analysis.

**Table S2:** Model 2–WLS results (detailed). Differences in healthcare experience among patients with self-declared diabetes. The chronic conditions' model.

| Variable | Category | Factor 1: INTER Coef. (CI 95%) | Factor 2: NEW Coef. (CI 95%) | Factor 3: SELF Coef. (CI 95%) | OVERALL IEXPAC Coef. (CI 95%) |
|---|---|---|---|---|---|
| Gender Baseline: Women | Hombre | -0.189 (-0.602,0.225) | 0.095 (-0.286,0.475) | -0.263 (-0.665,0.138) | -0.139 (-0.465,0.187) |
| Age ranges Baseline: 25-44 | 45 to 64 | 0.290 (-3.651,4.232) | 2.262* (-0.374,4.899) | 3.737** (0.697,6.777) | 2.082 (-0.616,4.779) |
| | 64 to 75 | 0.325 (-3.319,3.969) | 1.557 (-0.885,3.998) | 4.356** (1.543,7.169) | 2.127* (-0.376,4.630) |
| | 75 to 89 | 0.184 (-3.628,3.996) | 1.632 (-0.873,4.136) | 4.103** (1.146,7.059) | 2.004 (-0.626,4.634) |
| | >=90 | -0.164 -3.097,2.769 | 0.172 -1.392,1.736 | 1.296 -0.476,3.067 | 0.459 -1.356,2.273 |
| Occupation Baseline: Managers I | Managers II | 1.188 (-1.703,4.079) | 5.956*** (2.478,9.433) | 5.081** (0.903,9.259) | 3.904** (0.944,6.864) |
| | Intermediate | -0.088 (-1.632,1.455) | -0.633 (-1.635,0.369) | -0.354 (-2.097,1.390) | -0.333 (-1.588,0.921) |
| | Semi-qualified | -2.270 (-9.272,4.733) | 6.264** (1.701,10.828) | -0.317 (-4.728,4.094) | 0.768 (-4.318,5.854) |
| | Non-qualified | 0.709 (-1.683,3.101) | 1.290 (-0.726,3.306) | 3.229** (0.941,5.516) | 1.784** (0.033,3.535) |
| | Secondary-lower | -2.825 (-7.989,2.339) | 2.892 (-3.106,8.890) | -1.803 (-5.037,1.431) | -0.894 (-5.058,3.269) |
| | Secondary-upper | -2.040 (-6.301,2.221) | -2.003 (-4.478,0.472) | -0.718 (-3.160,1.724) | -1.549 (-4.551,1.453) |
| | Tertiary | 0.576 (-2.434,3.586) | 1.353 (-1.538,4.245) | 1.203 (-2.567,4.973) | 1.016 (-1.855,3.888) |
| | Managers II # 45 to 64 | -1.264 (-4.515,1.986) | -5.561** (-9.473,-1.649) | -4.193* (-8.598,0.212) | -3.501** (-6.700,-0.302) |
| | Managers II # 64 to 75 | -1.534 (-4.702,1.635) | -5.592** (-9.614,-1.571) | -5.174** (-9.561,-0.787) | -3.964** (-7.183,-0.746) |
| | Managers II # 75 to 89 | 0.649 (-2.760,4.059) | -6.117** (-10.557,-1.677) | -2.681 (-7.303,1.941) | -2.407 (-5.757,0.943) |
| | Managers II # >=90 | 1.187 (-1.013,3.386) | -5.138** (-8.222,-2.055) | 0.667 (-3.107,4.441) | -0.727 (-3.329,1.875) |
| | Intermediate # 45 to 64 | -1.591 (-5.094,1.912) | 0.838 (-2.113,3.789) | -0.356 (-2.813,2.101) | -0.480 (-3.091,2.132) |
| | Intermediate # 64 to 75 | -1.057 (-3.290,1.176) | 0.576 (-1.048,2.201) | -0.523 (-2.777,1.730) | -0.418 (-2.165,1.330) |
| | Semi-qualified # 45 to 64 | 2.162 (-4.922,9.246) | -6.217** (-11.000, -1.434) | 0.969 (-3.568,5.506) | -0.557 (-5.714,4.600) |
| | Semi-qualified # 64 to 75 | 1.853 (-5.180,8.886) | -5.869** (-10.546, -1.192) | 0.199 (-4.339,4.737) | -0.854 (-5.998,4.290) |
| | Semi-qualified # 75 to 89 | 2.381 (-4.715,9.477) | -6.353** (-11.017, -1.689) | 0.222 (-4.407,4.850) | -0.786 (-5.976,4.404) |
| | Semi-qualified # >=90 | 0.831 (-7.210,8.872) | -5.374** (-9.736, -1.012) | 2.160 (-3.897,8.217) | -0.378 (-6.265,5.509) |
| | Non-qualified # 45 to 64 | -1.568 (-4.301,1.166) | -1.449 (-3.773,0.874) | -3.549** (-6.194, -0.905) | -2.256** (-4.288, -0.224) |
| | Non-qualified # 64 to 75 | -1.238 (-3.638,1.162) | -1.146 (-3.331,1.040) | -3.851** (-6.229, -1.472) | -2.163** (-3.971, -0.355) |
| | Non-qualified # 75-89 | -0.969 (-3.616,1.679) | -1.303 (-3.551,0.946) | -3.628** (-6.185, -1.072) | -2.027** (-4.021, -0.033) |
| Education#Age Baseline: | Secondary-lower # 45 to 64 | 2.253 (-3.080,7.586) | -3.415 (-9.547,2.717) | 1.249 (-2.224,4.722) | 0.342 (-3.949,4.633) |

| | | | | | |
|---|---|---|---|---|---|
| Primary*25 to 44 | Secondary-lower # 64 to 75 | 2.687 (-2.543,7.917) | -2.757 (-8.790,3.276) | 1.798 (-1.520,5.116) | 0.879 (-3.334,5.092) |
| | Secondary-lower # 75 to 89 | 3.088 (-2.134,8.310) | -2.708 (-8.730,3.315) | 2.236 (-1.070,5.541) | 1.197 (-3.002,5.397) |
| | Secondary-lower # >=90 | 2.245 (-3.071,7.561) | -4.001 (-10.167,2.165) | 0.542 (-3.376,4.460) | -0.078 (-4.389,4.234) |
| | Secondary-upper # 45 to 64 | 1.755 (-2.708,6.218) | 1.784 (-0.831,4.399) | 0.587 (-2.142,3.316) | 1.338 (-1.822,4.498) |
| | Secondary-upper # 64 to 75 | 2.107 (-2.223,6.436) | 2.335* (-0.278,4.948) | 0.537 (-2.025,3.099) | 1.598 (-1.473,4.669) |
| | Secondary-upper # 75 to 89 | 2.402 (-1.942,6.746) | 3.022** (0.325,5.718) | 1.419 (-1.226,4.065) | 2.214 (-0.901,5.328) |
| | Secondary-upper # >=90 | -0.159 (-5.040,4.723) | 2.057 (-1.207,5.321) | -2.577 (-6.120,0.965) | -0.434 (-4.002,3.134) |
| | Tertiary # 45 to 64 | -0.397 (-3.738,2.944) | -0.998 (-4.334,2.338) | -1.334 (-5.303,2.635) | -0.902 (-3.987,2.184) |
| | Tertiary # 64 to 75 | -1.325 (-4.808,2.158) | -0.278 (-3.749,3.193) | -2.327 (-6.488,1.833) | -1.404 (-4.622,1.814) |
| | Tertiary # 75 to 89 | -1.736 (-5.246,1.774) | -0.378 (-4.006,3.251) | -1.868 (-6.215,2.478) | -1.414 (-4.741,1.914) |
| Chronic conditions | Caries | 0.500 (-0.484,1.484) | 0.098 (-0.756,0.953) | 0.576 (-0.350,1.503) | 0.418 (-0.325,1.161) |
| | Other, mouth | -0.040 (-0.834,0.754) | 0.088 (-0.617,0.793) | -0.113 (-1.128,0.903) | -0.032 -0.688,0.625 |
| | Hypertension | 0.067 (-0.317,0.451) | 0.162 (-0.185,0.510) | -0.069 (-0.457,0.320) | 0.044 (-0.266,0.353) |
| | Cholesterol (high) | -0.038 (-0.427,0.351) | -0.079 (-0.458,0.301) | -0.197 (-0.577,0.183) | -0.107 (-0.423,0.209) |
| | Fibromyalgia | 0.477 (-1.680,2.634) | 1.190* (-0.063,2.443) | 0.312 (-1.290,1.915) | 0.611 (-0.759,1.982) |
| | Rheumatism | 0.213 (-0.450,0.876) | -0.297 (-0.769,0.174) | 0.359 (-0.277,0.996) | 0.127 (-0.380,0.634) |
| | Upper-back pain | 0.060 (-0.611,0.732) | -0.477 (-1.048,0.095) | -0.201 (-0.920,0.518) | -0.181 (-0.719,0.357) |
| | Lower-back pain | -0.008 (-0.765,0.748) | -0.397 (-0.900,0.106) | -0.013 (-0.717,0.691) | -0.116 (-0.671,0.439) |
| | Asthma | -0.121 (-1.071,0.828) | -0.177 (-1.032,0.679) | 0.209 (-0.553,0.971) | -0.016 (-0.720,0.687) |
| | Varicose veins (in legs) | 0.239 (-0.341,0.819) | 0.091 (-0.473,0.655) | 0.024 (-0.606,0.654) | 0.121 (-0.350,0.591) |
| | Thrombosis | -0.127 (-1.307,1.052) | 0.029 (-1.105,1.163) | -0.252 (-1.395,0.891) | -0.130 (-1.074,0.814) |
| | AMI | -0.252 (-1.314,0.810) | -0.273 (-1.121,0.575) | -0.084 (-1.253,1.085) | -0.197 (-0.965,0.572) |
| | Other, heart | 0.112 (-0.395,0.619) | 0.188 (-0.288,0.664) | 0.261 (-0.273,0.795) | 0.187 (-0.229,0.603) |
| | Allergy | -0.608 (-2.668,1.451) | -0.524 (-1.393,0.346) | -0.572 (-2.156,1.011) | -0.572 (-1.968,0.824) |
| | COPD | -0.432 (-1.603,0.740) | -0.512 (-1.424,0.400) | -0.049 (-0.959,0.862) | -0.314 (-1.179,0.550) |
| | Stomach ulcer | -0.468 (-2.027,1.091) | -0.373 (-1.953,1.208) | -1.035 (-2.547,0.478) | -0.648 (-2.003,0.706) |
| | Hemorrhoids | -0.311 (-1.346,0.724) | 0.107 (-0.751,0.964) | -0.255 (-1.310,0.800) | -0.177 (-0.893,0.540) |
| | Cancer | -0.233 (-1.294,0.828) | 0.193 (-0.657,1.044) | 0.035 (-0.981,1.050) | -0.019 (-0.871,0.832) |
| | Blindness | -0.997 | 0.023 | -0.306 | -0.468 |

| | | | | | |
|---|---|---|---|---|---|
| | | (-2.484,0.489) | (-0.762,0.808) | (-1.727,1.116) | (-1.567,0.632) |
| | Cataracts | -0.144 | 0.053 | -0.191 | -0.108 |
| | | (-0.857,0.569) | (-0.665,0.770) | (-1.031,0.649) | (-0.720,0.505) |
| | Deafness | -0.195 | -0.212 | 0.007 | -0.127 |
| | | (-0.948,0.557) | (-0.709,0.285) | (-0.791,0.805) | (-0.701,0.448) |
| | Skin | -0.195 | 0.150 | 0.283 | 0.073 |
| | | (-1.526,1.137) | (-0.743,1.043) | (-0.850,1.417) | (-0.924,1.070) |
| | Thyroids | -0.126 | 0.270 | 0.127 | 0.074 |
| | | (-0.767,0.514) | (-0.318,0.859) | (-0.531,0.785) | (-0.440,0.588) |
| | Prostatitis | 0.148 | -0.440 | -0.177 | -0.130 |
| | | (-0.545,0.841) | (-1.117,0.238) | (-1.042,0.688) | (-0.765,0.504) |
| | Incontinence | 0.032 | -0.337 | -0.351 | -0.208 |
| | | (-0.834,0.899) | (-1.054,0.380) | (-1.333,0.630) | (-0.880,0.464) |
| | Dementia | 0.908 | 0.642 | 0.405 | 0.652 |
| | | (-0.244,2.060) | (-0.223,1.507) | (-1.047,1.856) | (-0.328,1.633) |
| | Anxiety | 0.256 | 0.670 | -0.045 | 0.259 |
| | | (-0.619,1.131) | (-0.383,1.724) | (-1.099,1.008) | (-0.548,1.067) |
| | Depression | 0.175 | -0.454 | 0.480 | 0.115 |
| | | (-0.653,1.003) | (-1.085,0.176) | (-0.439,1.400) | (-0.493,0.722) |
| | Other, mental | -0.862 | -0.919** | -0.443 | -0.725 |
| | | (-2.109,0.385) | (-1.734,-0.105) | (-2.257,1.371) | (-1.779,0.328) |
| | Migraine | -0.747 | -0.485 | -1.164 | -0.827 |
| | | (-2.074,0.579) | (-1.113,0.143) | (-2.738,0.411) | (-1.894,0.240) |
| | Osteoporosis | -0.124 | -0.447 | -0.731 | -0.433 |
| | | (-1.026,0.778) | (-1.047,0.154) | (-1.717,0.256) | (-1.112,0.246) |
| | Anemia | 0.873* | -0.144 | 0.418 | 0.430 |
| | | (0.088,1.658) | (-0.954,0.667) | (-0.482,1.319) | (-0.231,1.091) |
| | Constipation | -1.161** | -0.142 | -1.423** | -0.978** |
| | | (-2.157, -0.165) | (-0.957,0.674) | (-2.443, -0.402) | (-1.707, -0.250) |
| | Diabetic foot | -0.326 | 1.163 | 0.181 | 0.265 |
| | | (-1.771,1.119) | (-0.285,2.612) | (-1.661,2.023) | (-1.057,1.586) |
| | Blood circulation | -0.134 | -0.003 | 0.005 | -0.048 |
| | | (-0.873,0.604) | (-0.486,0.481) | (-0.673,0.682) | (-0.589,0.493) |
| | Insomnia | -0.594 | -0.170 | -0.329 | -0.382 |
| | | (-1.501,0.312) | (-0.684,0.343) | (-1.122,0.465) | (-1.019,0.255) |
| | Kidney | -0.743 | -0.148 | -0.713 | -0.570 |
| | | (-1.736,0.250) | (-0.813,0.517) | (-1.654,0.228) | (-1.312,0.172) |
| | Other chronic | 0.567 | -0.120 | 0.467 | 0.343 |
| | | (-0.309,1.442) | (-0.857,0.616) | (-0.354,1.289) | (-0.290,0.976) |
| | Constant | 8.546*** | -0.560 | 3.770** | 4.326*** |
| | | (4.906,12.186) | (-2.909,1.788) | (1.055,6.485) | (1.865,6.786) |
| Goodness of fit | R-squared | 0.124 | 0.147 | 0.140 | 0.123 |
| | BIC | 2740.861 | 2664.738 | 2749.151 | 2507.772 |
| Sample size (¥) | N | 554 | 554 | 554 | 554 |

i: Acute Myocardial Infarction; COPD: Chronic Obstructive Pulmonary Disorder; * $p < 0.1$, ** $p < 0.05$, *** $p < 0.001$; BIC: Bayesian Information Criterion; The sented model is corrected from heteroscedasticity using Eicker–Huber–White standard errors. ¥: There is one missing response for occupation, and has been uded for the analysis.

**Table S3.** Model 3–WLS results (detailed). Differences in healthcare experience among patients with diabetes. The effect of the presence of multi-morbidity.

| Variable | Category | Factor 1: INTER Coef. (C.I. 95%) | Factor 2: NEW Coef. (C.I. 95%) | Factor 3: SELF Coef. (C.I. 95%) | OVERALL IEXPAC Coef. (C.I. 95%) |
|---|---|---|---|---|---|

| | | | | | |
|---|---|---|---|---|---|
| Gender Baseline: Women | Men | -0.158<br>(-0.536,0.220) | 0.141<br>(-0.214,0.496) | -0.223<br>(-0.602,0.157) | -0.100<br>(-0.405,0.204) |
| Age ranges Baseline: 25 to 44 | 45 to 64 | -7.575***<br>(-11.329, -3.822) | -8.357***<br>(-12.602, -4.111) | -6.026***<br>(-9.594, -2.458) | -7.225***<br>(-10.291, -4.158) |
| | 64 to 75 | -7.894***<br>(-11.396, -4.393) | -8.669***<br>(-12.849, -4.489) | -5.607**<br>(-8.991, -2.222) | -7.274***<br>(-10.196, -4.352) |
| | 75 to 89 | -8.445***<br>(-12.154, -4.735) | -9.081***<br>(-13.239, -4.922) | -6.145**<br>(-9.810, -2.481) | -7.782***<br>(-10.832, -4.732) |
| | >=90 | 0.255<br>(-2.952,3.461) | -3.717***<br>(-5.791, -1.644) | -1.244<br>(-4.388,1.901) | -1.374<br>(-3.784,1.037) |
| Education Baseline: Primary | Secondary-lower | -10.690***<br>(-11.626, -9.753) | -5.331**<br>(-8.586, -2.076) | -11.017***<br>(-11.675, -10.359) | -9.347***<br>(-10.707, -7.988) |
| | Secondary-upper | -10.876***<br>(-11.753, -9.999) | -11.054***<br>(-14.305, -7.803) | -10.678***<br>(-11.244, -10.112) | -10.853***<br>(-12.190, -9.515) |
| | Tertiary | -0.266<br>(-0.832,0.300) | -0.653<br>(-2.529,1.223) | -0.710***<br>(-0.981, -0.439) | -0.533<br>(-1.326,0.261) |
| Occupation Baseline: Managers I | Managers II | 5.632**<br>(1.112,10.152) | 8.280***<br>(6.207,10.354) | 8.836***<br>(5.399,12.273) | 7.519***<br>(4.589,10.449) |
| | Intermediate | 0.174<br>(-1.072,1.420) | -0.754<br>(-1.625,0.118) | -0.004<br>(-1.467,1.459) | -0.143<br>(-1.131,0.844) |
| | Semi-qualified | 4.364**<br>(1.099,7.628) | 8.130***<br>(4.777,11.483) | 4.296**<br>(1.125,7.468) | 5.366***<br>(2.737,7.996) |
| | Non-qualified | 3.020*<br>(-0.133,6.174) | 1.102**<br>(0.220,1.984) | 4.466**<br>(1.335,7.597) | 3.023**<br>(0.747,5.298) |
| Occupation # Age | Managers II # 45 to 64 | -5.712**<br>(-10.380, -1.045) | -7.653***<br>(-10.380, -4.926) | -7.940***<br>(-11.559, -4.321) | -7.051***<br>(-10.150, -3.953) |
| | Managers II # 64 to 75 | -6.272**<br>(-11.070, -1.474) | -7.620***<br>(-10.399, -4.841) | -8.962***<br>(-12.720, -5.204) | -7.618***<br>(-10.854, -4.382) |
| | Managers II # 75 to 89 | -3.975*<br>(-8.658,0.708) | -8.422***<br>(-11.973, -4.871) | -6.602***<br>(-10.316, -2.887) | -6.143***<br>(-9.323, -2.962) |
| | Managers II # >=90 | -3.132<br>(-7.652,1.388) | -8.280***<br>(-10.354, -6.207) | -3.836**<br>(-7.273, -0.399) | -4.792**<br>(-7.722, -1.862) |
| | Intermediate # 45 to 64 | -1.804<br>(-4.982,1.373) | 1.125<br>(-1.254,3.505) | -0.594<br>(-2.729,1.540) | -0.565<br>(-2.820,1.689) |
| | Intermediate # 64 to 75 | -1.354<br>(-3.307,0.600) | 0.771<br>(-0.582,2.124) | -0.731<br>(-2.713,1.251) | -0.548<br>(-2.034,0.939) |
| | Semi-qualified # 45 to 64 | -4.364**<br>(-7.785, -0.943) | -8.101***<br>(-11.709, -4.494) | -3.612**<br>(-6.975, -0.250) | -5.110***<br>(-7.890, -2.329) |
| | Semi-qualified # 64 to 75 | -4.739**<br>(-8.130, -1.347) | -7.661***<br>(-11.159, -4.163) | -4.224**<br>(-7.532, -0.916) | -5.348***<br>(-8.088, -2.609) |
| | Semi-qualified# 75 to 89 | -3.990**<br>(-7.371, -0.608) | -7.944***<br>(-11.412, -4.477) | -4.084**<br>(-7.472, -0.695) | -5.102***<br>(-7.849, -2.356) |
| | Semi-qualified # >=90 | -3.660<br>(-8.047,0.728) | -7.133***<br>(-10.566, -3.699) | -1.678<br>(-5.886,2.530) | -3.886**<br>(-7.239, -0.533) |
| | Non-qualified # 45 to 64 | -3.900**<br>(-7.238, -0.563) | -1.251*<br>(-2.667,0.165) | -4.736**<br>(-8.054, -1.418) | -3.482**<br>(-5.932, -1.032) |
| | Non-qualified # 64 to 75 | -3.632**<br>(-6.893, -0.372) | -0.845<br>(-2.076,0.386) | -4.956**<br>(-8.185, -1.728) | -3.354**<br>(-5.719, -0.989) |
| | Non-qualified # 75 to 89 | -3.159*<br>(-6.431,0.112) | -0.999<br>(-2.195,0.198) | -4.672**<br>(-7.992, -1.353) | -3.120<br>(-5.517, -0.724) |

| | | | | | |
|---|---|---|---|---|---|
| Education#Age | Secondary-lower # 45 to 64 | 9.970*** | 4.788** | 10.510*** | 8.753*** |
| | | (8.424,11.516) | (1.339,8.238) | (9.192,11.827) | (7.094,10.412) |
| | Secondary-lower # 64 to 75 | 10.273*** | 5.202** | 10.702*** | 9.046*** |
| | | (9.070,11.475) | (1.900,8.504) | (9.725,11.679) | (7.565,10.527) |
| | Secondary-lower # 75 to 89 | 10.652*** | 5.301** | 11.006*** | 9.321*** |
| | | (9.505,11.799) | (1.992,8.610) | (10.030,11.983) | (7.855,10.787) |
| | Secondary-lower # >=90 | 9.976*** | 3.904** | 9.276*** | 8.065*** |
| | | (7.505,12.448) | (0.524,7.284) | (7.109,11.442) | (6.057,10.074) |
| | Secondary-upper # 45 to 64 | 10.376 | 10.869 | 10.473 | 10.546 |
| | | (8.763,11.989) | (7.460,14.279) | (9.165,11.781) | (8.881,12.211) |
| | Secondary-upper # 64-75 | 10.859*** | 11.368*** | 10.446*** | 10.848*** |
| | | (9.740,11.979) | (8.015,14.721) | (9.495,11.398) | (9.371,12.325) |
| | Secondary-upper # 75-89 | 11.028*** | 11.965*** | 11.057*** | 11.294*** |
| | | (9.864,12.192) | (8.523,15.407) | (9.862,12.252) | (9.732,12.857) |
| | Secondary-upper # >=90 | 8.843*** | 12.028*** | 7.955*** | 9.389*** |
| | | (8.018,9.668) | (8.794,15.262) | (7.473,8.438) | (8.075,10.703) |
| | Tertiary # 45 to 64 | 0.292 | 1.086 | 0.499 | 0.584 |
| | | (-1.230,1.815) | (-1.344,3.516) | (-0.712,1.710) | (-0.756,1.924) |
| | Tertiary # 64 to 75 | -0.436 | 1.623 | -0.355 | 0.155 |
| | | (-2.290,1.417) | (-0.918,4.164) | (-2.151,1.441) | (-1.458,1.768) |
| | Tertiary # 75 to 89 | -0.861 | 1.474 | 0.022 | 0.097 |
| | | (-2.796,1.075) | (-1.413,4.361) | (-2.041,2.086) | (-1.707,1.901) |
| Number of conditions. Baseline: 1 | 2 | -12.073*** | -10.657*** | -11.372*** | -11.432*** |
| | | (-15.428, -8.717) | (-14.591, -6.724) | (-12.930, -9.814) | (-13.743, -9.121) |
| | 3 | -11.336*** | -10.146*** | -12.627*** | -11.481*** |
| | | (-12.431, -10.242) | (-15.395, -4.896) | (-13.313, -11.942) | (-13.539, -9.423) |
| | +3 | -9.461*** | -5.979** | -7.002*** | -7.617*** |
| | | (-10.556, -8.367) | (-11.229, -0.729) | (-7.688, -6.317) | (-9.675, -5.559) |
| Age#Number of conditions | 45 to 64 # 2 | 11.715*** | 10.711*** | 11.281*** | 11.283*** |
| | | (8.174,15.257) | (6.689,14.733) | (9.353,13.209) | (8.819,13.748) |
| | 45 to 64 # 3 | 10.677*** | 10.804*** | 12.542*** | 11.390*** |
| | | (9.043,12.311) | (5.429,16.179) | (11.178,13.906) | (9.125,13.654) |
| | 45 to 64 # +3 | 8.794*** | 6.482** | 6.753*** | 7.421*** |
| | | (7.176,10.412) | (1.150,11.813) | (5.390,8.116) | (5.168,9.675) |
| | 64 to 75 # 2 | 11.839*** | 11.390*** | 11.600*** | 11.629*** |
| | | (8.318,15.359) | (7.304,15.475) | (9.708,13.492) | (9.135,14.124) |
| | 64 to 75 # 3 | 11.015*** | 10.052*** | 12.499*** | 11.292*** |
| | | (9.572,12.459) | (4.716,15.389) | (11.315,13.683) | (9.081,13.503) |
| | 64 to 75 # +3 | 9.721*** | 5.471** | 6.993*** | 7.570*** |
| | | (8.311,11.132) | (0.146,10.796) | (5.846,8.139) | (5.372,9.768) |
| | 75 to 89 # 2 | 12.397*** | 11.877*** | 12.207*** | 12.186*** |
| | | (8.715,16.079) | (7.791,15.963) | (10.021,14.392) | (9.600,14.772) |
| | 75 to 89 # 3 | 11.301*** | 10.455*** | 12.615*** | 11.548*** |
| | | (9.359,13.243) | (5.137,15.773) | (10.890,14.340) | (9.174,13.922) |
| | 75 to 89 # +3 | 9.480*** | 5.993** | 6.955*** | 7.611*** |
| | | (7.635,11.324) | (0.686,11.299) | (5.349,8.562) | (5.281,9.940) |
| | >=90 # 2 | -0.837 | 3.752*** | 1.130 | 1.130 |
| | | (-6.832,5.159) | (1.687,5.818) | (-3.961,6.222) | (-2.902,5.163) |
| | >=90 # 3 | -0.555 | 5.099*** | 3.481** | 2.454** |
| | | (-2.881,1.770) | (4.118,6.079) | (1.372,5.590) | (0.937,3.972) |
| Constant | | 16.865*** | 9.556*** | 13.469*** | 13.636*** |
| | | (13.512,20.217) | (5.502,13.610) | (10.230,16.707) | (10.832,16.441) |
| Goodness-of-fit | R-squared | 0.128 | 0.156 | 0.120 | 0.123 |
| | BIC | 2580.621 | 2488.809 | 2604.232 | 2343.248 |

| | | | | | |
|---|---|---|---|---|---|
| Heteroscedasticity correction method | YES | Robust variance | Robust variance | Robust variance | Robust variance |
| | N | 554.000 | 554.000 | 554.000 | 554.000 |

* $p < 0.1$, ** $p < 0.05$, *** $p < 0.001$; BIC: Bayesian Information Criterion; The presented model is corrected from heteroscedasticity using Eicker–Huber–White standard errors. ¥: There is one missing response for occupation, and has been excluded for the analysis.
